# A rare variant of mullerian agenesis: a case report and review of the literature

**DOI:** 10.1186/s13256-024-04438-x

**Published:** 2024-03-25

**Authors:** Shriya Devendra Tayade, Nadia Mehdi, Rajani Dube, Vaishnavi Bose, Ashna Ameer, Zainabsadat Tabatabaei Hakim, Arnaud Wattiez

**Affiliations:** 1 Intern, RAK Medical and Health Sciences University, Ras Al Khaimah, UAE; 2 Emirates Health Service, Dubai, UAE; 3 Obstetrics and Gynecology, RAK Medical and Health Sciences University, Ras Al Khaimah, UAE; 4 Obstetrics and Gynecology, Specialist in Minimally Invasive Surgery, Latifa Women and Children Hospital, Dubai, UAE; 5 Gynecology and Minimally Invasive Surgery, Department of Gynecology, Latifa Women and Children Hospital, Dubai, UAE

**Keywords:** Mullerian agenesis, Mayer–Rokitansky–Kuster–Hauser syndrome, Case report

## Abstract

**Introduction:**

Menstruation is a developmental milestone and usually marks healthy and normal pubertal changes in females. Menarche refers to the onset of first menstruation in a female. The causes of primary amenorrhea include outflow tract abnormalities, resistant endometrium, primary ovarian insufficiency, and disorders of the hypothalamus, pituitary, or other endocrine glands. A rare variant of mullerian agenesis, which warrants an individualized approach to management, is presented here.

**Case report:**

We present here the case of a 25-year-old Indian female with pain in the lower abdomen and primary amenorrhea. After a thorough history, clinical examination, imaging, and diagnostic laparoscopy, two small uteri, a blind upper half vagina, bilateral polycystic ovaries, and a blind transverse connection between the two uteri—a horseshoe band cervix—were detected, which confirmed the diagnosis of mullerian agenesis. There was evidence of adenomyosis in the mullerian duct element. This is a rare form of Müllerian abnormality with an unusual presentation.

**Conclusion:**

Mullerian agenesis is the most common cause of primary amenorrhea with well-developed secondary sexual characteristics. There are various forms of mullerian agenesis. Most of the cases are managed by a multidisciplinary team. Rare variants warrant an individualized approach to management.

## Introduction

Menstruation is a developmental milestone and usually marks healthy and normal pubertal changes in females. Menarche refers to the onset of first menstruation in a female. Amenorrhea is the absence of menses and can be primary or secondary. In primary amenorrhea (PA), there is an absence of menarche, whereas secondary amenorrhea (SA) refers to the cessation of previously regular menses for 3 months or irregular menses for 6 months [1]. According to the American College of Obstetricians and Gynecologists (ACOG), the evaluation of PM should be started if there is no menstruation by 15 years of age or 3 years after the onset of pubertal changes such as thelarche [1, 2]. Delayed puberty should be suspected and investigated if there is a lack of any pubertal development by 13 years of age [3].

The causes of PA include outflow tract abnormalities, resistant endometrium, primary ovarian insufficiency, and disorders of the hypothalamus, pituitary, or other endocrine glands. The most common cause of PA is gonadal dysgenesis, followed by mullerian duct agenesis [4]. While in gonadal dysgenesis, the secondary sexual characteristics are not well developed owing to a lack of estrogen production by dysgenetic (streak) ovaries, mullerian duct abnormalities with outflow tract obstruction should be suspected in females with otherwise well-developed secondary sexual characteristics. Müllerian agenesis (mullerian aplasia, vaginal agenesis) or Mayer–Rokitansky–Küster–Hauser syndrome is rare, with an incidence of 1 per 4500–5000 females [5]. Although chronic illnesses and iatrogenic causes are mostly attributable to SA, some of these pathologies present early in life and present as PA [1, 6–10].

Here, we present a 25-year-old female with pain in the lower abdomen and PA. After a thorough history, clinical examination, imaging, and diagnostic laparoscopy, two small uteri, a blind upper half vagina, bilateral polycystic ovaries, and a blind transverse connection between the two uteruses—horseshoe band cervix—were detected, which confirmed the diagnosis of Mayer–Rokitansky–Kuster–Hauser (MRKH) syndrome. This is a rare form of Müllerian abnormality with an unusual presentation.

## Case report

A 25-year-old Indian female presented to the emergency department with severe abdominal pain for 2 days. She was in distress owing to pain but did not complain of associated fever, nausea, vomiting, bowel disturbances, such as diarrhea or constipation, or any urinary symptoms. The severity of the pain was 9 (on a scale of 1–10).

Her pubertal development review revealed the onset of breast enlargement (thelarche) at the age of 11 and pubic hair development (pubarche) at the age of 12. She was born at term by vaginal delivery of an uncomplicated pregnancy. There was no history of exposure to radiation, specific medications, or infection in her mother during pregnancy. The developmental history for motor, sensory, and social milestones was normal. The rest of her history, including medical, surgical, and social history, was unremarkable. She was on analgesics occasionally but not on any long-term medications. She was unmarried and had never been sexually active. There was no family history of any congenital anomalies among the family members. Further questioning revealed that she had experienced irregular abdominal pain described as “dull and cramping,” since the age of 13. By the age of 16, the pain had become more or less cyclic at intervals of 45–50 days. All the episodes of pain were bearable, infrequent, and treated by analgesics as and when required. There was never an episode of urinary retention, a need for injectable analgesics, or hospital admission.

On examination, observations were normal. She was of average height, and weight, with a normal arm span and normal body mass index. She was phenotypically female with normal breast and pubic hair development (Tanner 4). Abdominal examination revealed tenderness in the right lower quadrant with no guarding or rigidity. Bowel sounds were present. A bimanual vaginal examination was not done.

**Fig. 1:**
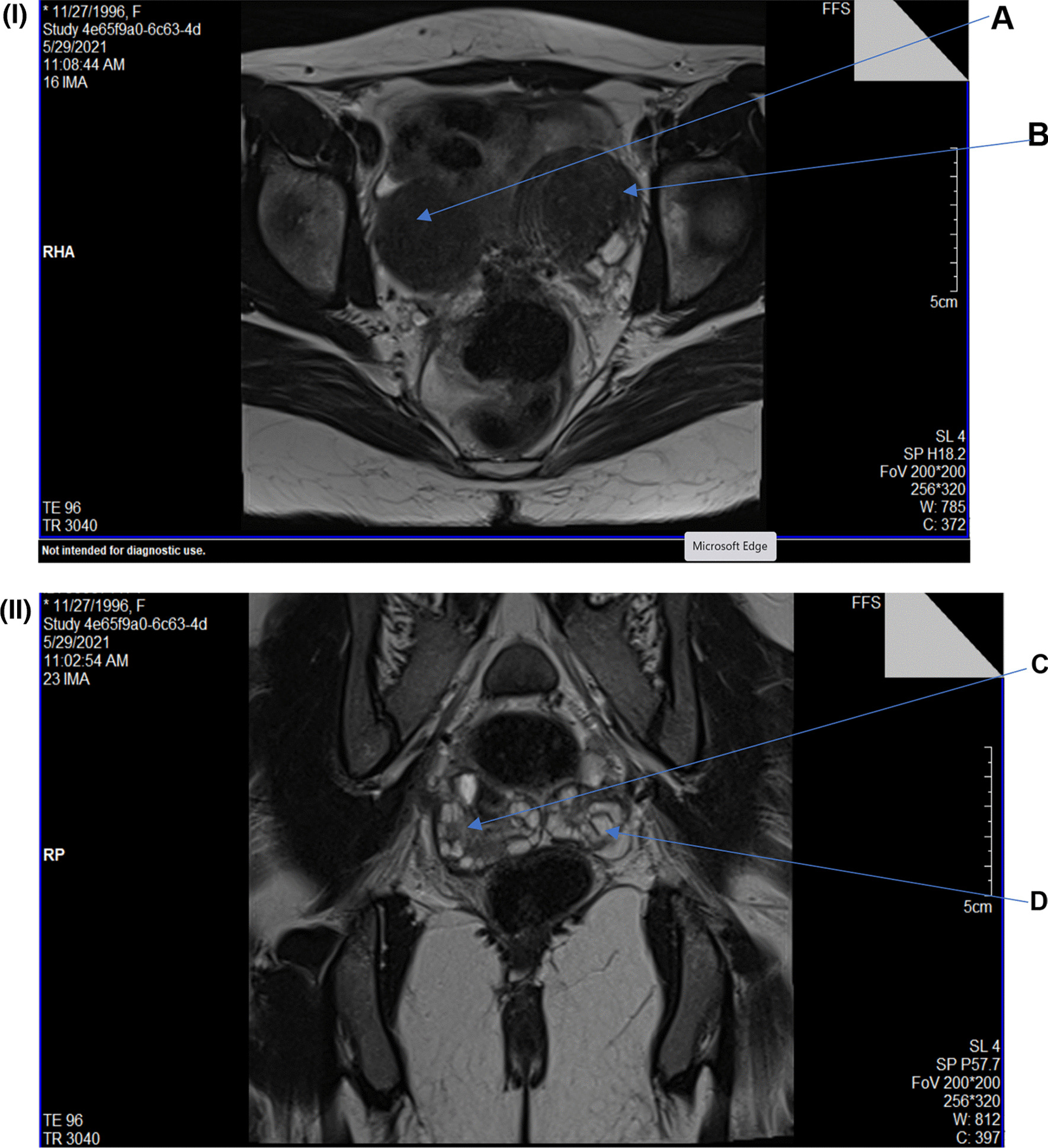
MRI of the pelvis: **A** Blue arrow shows uterine cavity of one cornue of bicornuate uterus. **B** Blue arrow shows heterogeneous myometrial signal intensity with a thickened junctional zone and indistinct endo-myometrial junction, likely adenomyosis. **C**, **D** Blue arrow shows both ovaries that were polycystic

**Fig. 2:**
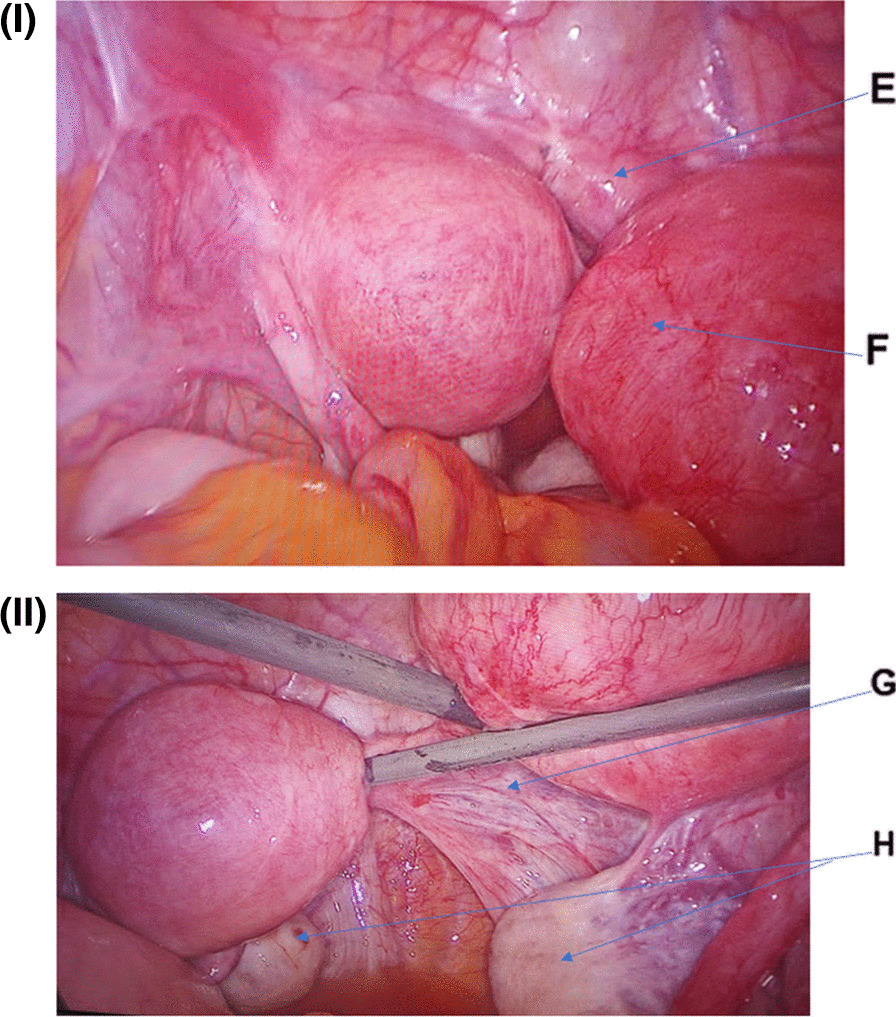
Diagnostic laparoscopy findings–the upper abdomen and appendix looked normal; both ureters are seen with peristalsis; two uteri can be seen; the right uterus has right round ligament and right fallopian tube; the left uterus has left round ligament and left fallopian tube; one single-blind transverse connection between the two uteruses (horseshoe band cervix) (**E**); the right uterus is bulkier than the left (**F**); there was no vaginal or any other connections (**G**); and both ovaries are polycystic looking and otherwise normal in position and anatomy (**H**)

An abdominal ultrasound (US) was done, which showed the presence of blood in the right uterus and multiple small ovarian cysts. There was no other abnormalities detected. Magnetic resonance imaging (MRI) without contrast revealed a bicornuate uterus (likely a unicollis), with both horns revealing features of adenomyosis. It also showed a single cervix that appeared angulated with suboptimal visualization of the uterocervical junction and a blind upper half of the vagina. There were polycystic-appearing ovaries with multiple small peripheral follicles and thickened stroma, placed posteriorly in the pouch of Douglas (Fig. 1  I, II). She was then advised to undergo a diagnostic laparoscopy, which ultimately showed the presence of two small uteruses, a blind upper half of the vagina, and bilateral ovaries with peripheral follicles. There was a blind transverse connection between the two uteruses and a horseshoe band cervix, which confirmed the diagnosis of mullerian agenesis or Mayer–Rokitansky–Kuster–Hauser (MRKH) syndrome (Fig. 2I, II). Laboratory investigations showed normal female values for testosterone, follicle stimulating hormone (FSH), luteinizing hormone (LH), and thyroid stimulating hormone (TSH). The karyotyping of this patient was that of a normal female, i.e., 46, XX. There were no morphological abnormalities in the kidneys and both kidneys were normally located. There were no detectable cardiac or vertebral abnormalities. Hence, it was confirmed to be type 1 MRKH syndrome.

She received IV parecoxib and extensive counseling from a consultant gynecologist regarding further management options and future implications. The treatment offered included medical, surgical, and future fertility options. Medical treatment consisted of continuous combined estrogen–progesterone pills or progesterone-only pills. The surgical options were vaginal reconstructive surgery with a possible connection to the right-side uterus with functional endometrium. Future fertility options included gestational surrogacy with own eggs, adoption, or hysterectomy followed by uterine transplant (UT), and pregnancy by *in vitro* fertilization. Counseling of her and her family members was done by the counselors to manage psychological distress. She was started on continuous oral contraceptive pills (OCPs) and was asked to follow up in the gynecology clinic as she opted for medical treatment. She agreed to regular follow-up and to undergo surgical treatment at a later date if needed. She is currently following up regularly, on progesterone-only pills, and without any further episodes of abdominal pain.

## Case summary

A 25-year-old unmarried Indian female presented to the emergency room with severe abdominal pain for 2 days without fever, vomiting, gastrointestinal, urinary, or any other associated systemic symptoms. She was hemodynamically stable, with mild tenderness in the right suprapubic region, but the rest of the examinations revealed no abnormalities. The past history was suggestive of PA, with well-developed secondary sexual characteristics and irregular abdominal pain. The rest of the history, including developmental, family, medical, surgical, and social, was normal. Investigation revealed a normal hemogram, a normal hormonal profile for an adult female, and a 46, XX karyotype. US and MRI revealed a rare variant of MRKH syndrome, polycystic ovaries, and adenomyotic changes in uterine elements. A diagnostic laparoscopy confirmed the mullerian abnormality. She was treated with analgesics and discharged with continuous OCPs as medical management. Psychological support and counseling were given, and surgical and fertility options were discussed. She is being followed up by a multidisciplinary team, is currently asymptomatic, and continuing medical management.

## Discussion

The differential diagnosis was PA with thelarche appropriate for age pointing to an obstructive etiology or complete androgen insensitivity syndrome (AIS). In MRKH syndrome, the individual is reared like a female with phenotypically normal external genitalia. They go on to develop normal secondary sexual characteristics with normal pubarche. However, they lack menstruation owing to the absence of a uterus or cervix or upper vagina, or a combination of all [11]. On vaginal examination or probing, there is usually a short, blind vaginal pouch (Table 1).

**Table 1:** Differential diagnosis of primary amenorrhea with developed secondary sexual characteristics

Parameters	Conditions			
	MRKH syndrome	AI Syndrome	Transverse vaginal septum	Imperforate hymen
Symptoms	Primary amenorrhea	Primary amenorrhea	Primary amenorrhea with cyclical abdominal pain urinary retention (rare)	Primary amenorrhea with cyclical abdominal pain urinary retention (rare)
Examination findings	Normal height external genitalia–normal female Thelarche–normal Pubarche–normal	Usually taller than normal females for their age. External genitalia–normal female, unilateral or bilateral mass in the inguinal canal thelarche–normal pubarche–sparse hair or absent	Normal height external genitalia–normal female Thelarche–normal Pubarche–normal	Normal height external genitalia–bluish, bulging membrane Thelarche–normal Pubarche–normal
Karyotype	46, XX	46, XY	46, XX	46, XX
Imaging	Absence of uterus, cervix, upper vagina, or all, normal ovaries	Absence of uterus, cervix, and upper vagina, absence of internal Wolffian duct elements, testicular tissue in the inguinal canal	Hematocolpos with or without hematometra empty lower vagina septa may be seen	Hematocolpos with or without hematometra collection up to the opening of the introitus
Hormonal levels	Normal FSH, LH testosterone–female levels	Normal FSH, LH testosterone–male levels	Normal FSH, LH testosterone–female levels	Normal FSH, LH testosterone–female levels

Individuals with complete AIS are also generally reared like a female as the external genitalia resemble a normal female. They can also show thelarche owing to the conversion of testosterone to estrogen. However, morphologically they show sparse or absent terminal hair in the pubic region and axilla. They may show a mass in the inguinal canal representing the testis unilaterally or bilaterally owing to the absence of a scrotum [12, 13]. On vaginal examination or probing there is usually a short blind vaginal pouch.

In both the imperforate hymen and transverse vaginal septum, there is an obstruction of the outflow tract in an individual with a normally functioning hypothalamic–pituitary–ovarian (HPO) axis and a normal uterus. They present with cyclical abdominal pain and PA. While the former presents with a bluish, bulging membrane at the introitus, the latter presents with an obstructed vaginal canal. In both conditions, there is a collection of menstrual blood in the vagina (hematocolpos) above the level of obstruction.

A detailed history and thorough examination help in the provisional diagnosis of specific conditions. Investigations are done for confirmation of the diagnosis and to formulate a management plan. MRI is recommended in patients to show the presence of the uterus or remnants, the presence or absence of gonads (testis or ovary), the location of the gonads, and the collection of blood [14, 15]. Karyotype helps to differentiate AIS (46, XY) from those with MRKH (46, XX). Assessment of serum levels of FSH, LH, and testosterone further helps in confirming AIS, wherein the testosterone levels will resemble those of a male individual.

The American Society for Reproductive Medicine (ASRM) Mullerian Anomalies Classification 2021 (MAC2021) divides it into nine categories while allowing for an overlap of abnormalities [11]. When the abnormalities of our patient were interpreted in the context of the MAC2021, the abnormalities belonged to mullerian agenesis (MA), right-side uterine remnant with functional endometrium, left-side remnant with nonfunctional endometrium, and cervical hypoplasia/agenesis. There was also adenomyosis in the remnant with functional endometrium, and bilateral polycystic ovaries. There were no other features of polycystic ovary syndrome [10]. Furthermore, patients with MA and hematocolpos usually present earlier in their teens. Hence, it is a rare combination of abnormalities and poses significant challenges for diagnosis as well as management. A previous cohort study of 284 women with MRKH reported various combinations for abnormalities but not in the combination seen in our patient [8]. Another review of 11 patients from a center also revealed bilateral uterine remnants in patients with MRKH syndrome [16]. However, it did not match these abnormalities. Patients with MRKH syndrome might very rarely present with inguinal hernia and associated complications [17]. However, hernial orifices were normal in our patient.

The diagnosis of MRKH syndrome poses psychological distress for the patient. Hence, treatment is normally done by a multidisciplinary team comprising gynecologists, fertility specialists, and psychological counselors. Support groups can also help the patient alleviate the associated stress [13]. Treatment for MRKH syndrome normally includes progressive vaginal dilators, surgical creation of a neovagina, or other complex procedures and should be referred to specialized centers [9, 12, 18]. Fertility options include adoption, surrogacy with the use of an ovum from the woman herself, or UT [6, 9, 19]. When biologically related offspring is planned (surrogacy, UT), genetic analysis should be done and inheritance risk should be considered [20]. Moreover, all the options may not be available in all countries owing to sociocultural or ethical issues [19].

## Conclusion

Mullerian agenesis is the most common cause of PA with well-developed secondary sexual characteristics. There are various forms of mullerian agenesis. Most of the cases are managed by a multidisciplinary team. Rare variants warrant an individualized approach to management.

## Disclaimer

The views expressed in the submitted article are our own and not an official position of the institution or funder.

## Data Availability

All the materials are available from the corresponding author and can be provided upon reasonable request.
